# PHLPP1 inhibits the growth and aerobic glycolysis activity of human ovarian granular cells through inactivating AKT pathway

**DOI:** 10.1186/s12905-023-02872-5

**Published:** 2024-01-06

**Authors:** Xiaoyan Yang, Min A, Tana Gegen, Badema Daoerji, Yue Zheng, Aiming Wang

**Affiliations:** 1 Reproductive Medicine Center, Affiliated Hospital of Inner Mongolia Minzu University, Tongliao, Inner Mongolia 028000 China; 2 Clinical Medical (Mongolian Medical) College of Inner Mongolia Minzu University, Tongliao, Inner Mongolia 028000 China; 3 Department of Urology, Affiliated Hospital of Inner Mongolia Minzu University, Tongliao, Inner Mongolia 028000 China; 4 Department of Obstetrics and Gynaecology, Sixth Medical Center, Chinese PLA General Hospital, No.6 Fucheng Road, Haidian District, Beijing, 100048 China

**Keywords:** PHLPP1, Infertility, Polycystic ovary syndrome, Glycolysis, AKT signaling

## Abstract

**Background:**

Polycystic ovary syndrome (PCOS) is a disorder characterized by hyperandrogenism, ovulatory dysfunction, and polycystic ovarian morphologic features, and PCOS is associated with infertility. PH domain Leucine-rich repeat Protein Phosphatase 1 (PHLPP1) has been shown to regulate AKT. The aim of present study is to investigate the role of PHLPP1 in PCOS.

**Methods:**

The expression levels of PHLPP1 in dihydrotestosterone (DHT)-treated human ovarian granular KGN cells were determined by qRT-PCR and Western blot. PHLPP1 was silenced or overexpressed using lentivirus. Cell proliferation was detected by CCK-8. Apoptosis and ROS generation were analyzed by flow cytometry. Glycolysis was analyzed by measuring extracellular acidification rate (ECAR).

**Results:**

DHT treatment suppressed proliferation, promoted apoptosis, enhanced ROS, and inhibited glycolysis in KGN cells. PHLPP1 silencing alleviated the DHT-induced suppression of proliferation and glycolysis, and promotion of apoptosis and ROS in KGN cells. PHLPP1 regulated cell proliferation and glycolysis in human KGN cells via the AKT signaling pathway.

**Conclusions:**

Our results showed that PHLPP1 mediates the proliferation and aerobic glycolysis activity of human ovarian granular cells through regulating AKT signaling.

## Background

Infertility is the inability to get pregnant after 1 year of regular intercourse [1]. Infertility has a 9 to 18% prevalence [2]. In USA, approximately 13% of reproductive age women seek treatment for infertility every year [3]. About 85% of cases are because of ovulatory dysfunction, male factor infertility, and tubal disease. The other 15% of cases have “unexplained infertility” [3]. Polycystic ovary syndrome (PCOS), a common metabolic disorder in premenopausal women, is characterized by hyperandrogenism, ovulatory dysfunction, and polycystic ovarian morphologic features [4, 5]. PCOS is a major cause of hyperandrogenism and infertility [6]. The prevalence of infertility in women with PCOS ranges from 70 to 80% [7]. PCOS affects 6–20% of reproductive aged women [8]. The etiology is still unclear, but data support that it may be a multigenic disorder with strong epigenetic influences [4]. Risk factors include genetics, neuroendocrine, lifestyle/environment, obesity [9]. Treatment of PCOS is individualized accordingly. For example, weight control should be considered for overweight patients, clomiphene is recommended for infertility [10]. Regardless of the advances in treating PCOS, more studies are needed for better understanding the underlying mechanisms in order to provide more effective and precise treatment.

Glycolysis metabolizes glucose to pyruvate and generates ATP in cytoplasm [11]. The 3 key enzymes for glycolysis are hexokinase, phosphofructokinase, and pyruvate kinase [12]. Glycolysis plays a very important role in various pathological processes. For instance, Lim et al. have reported that inhibiting glycolysis and EGFR effectively suppressed mammary cancer growth [13]. Xiang et al. demonstrated that enhanced glycolysis in skeletal muscle coordinates with adipose tissue in systemic metabolic homeostasis [14]. Glycolysis is also essential for sperm motility and male fertility because sperm require ATP that is produced by mitochondrial oxidative phosphorylation and glycolysis to provide energy for motility [15]. Aberrant expression of glycolytic enzymes has been shown to be associated with infertility [16]. Glycolysis also involves in PCOS. For example, Zhao et al. have demonstrated that elevated glycolysis and decreased tricarboxylic acid cycle were presented in women with PCOS [17]. Dysregulation of glycolysis has also been associated with PCOS patients with hyperplasia [18]. Despite the remarkable success of the study of glycolysis, the exact role of glycolysis in PCOS and how glycolysis is involved remain unclear and need to be elucidated.

PH domain Leucine-rich repeat Protein Phosphatase 1 (PHLPP1) is a new member of the phosphatome [19]. PHLPP1 was first discovered by Gao et al. for its inhibiting growth factor signaling through dephosphorylation of AKT (protein kinase B, PKB) [20]. Dysregulation of PHLPP involves in different pathophysiology, from cancer to metabolic diseases [21]. Moc et al. have reported that physiological activation of AKT signaling by PHLPP1 deletion protects against pathological hypertrophy [22]. In contrast, overexpressing PHLPP1 decreases Akt and suppresses melanoma cell growth [23]. It is known that the phosphoinositide 3-kinase-AKT pathway regulates glycolysis [24]. AKT activation leads to promotion of aerobic glycolysis and overexpressing AKT leads to increased glycolytic rate [25, 26]. As mentioned above, dysregulated glycolysis plays a crucial role in PCOS. However, the role of PHLPP1/AKT/glycolysis in infertility and PCOS is not clear and remains to be elucidated.

Therefore, how PHLPP1 regulates glycolysis and how this PHLPP1-regulated-glycolysis affects human ovarian granular cell proliferation were investigated in this study to provide scientific basis for developing new treatments for PCOS.

## Methods

### Cell culture and treatment

The human ovarian granular KGN cells from Shanghai Biology Institute were cultured in DMEM with 10% FBS, 2 mM l-glutamine (Biyuntian, Suzhou) and antibiotics (100 IU/mL penicillin, 100 µg/mL streptomycin) (Sigma, Saint Louis, MO, USA) under 37 °C. Cells were treated with 25 nM DHT (Abmole Bioscience Inc., Houston, TX, USA) with or without PHLPP1 knockdown, followed by 5 µM MK-2206 (an AKT inhibitor; Selleck, Shanghai, China) or not. Otherwise, cells with PHLPP1 overexpression were treated with 5 µg/mL SC79 (an AKT activator; Selleck).

### qRT-PCR

Total RNA was isolated by TRIzol, reverse-transcribed to synthesize cDNA utilizing PrimeScript RT Reagent Kit (Takara Biomedical Technology (Beijing) Co., Ltd.). qRT-PCR was performed using Maxima SYBR Green/ROX qPCR (Thermo Fisher Scientific). Fold change was calculated by 2^−ΔΔCt^. Primers (5’-3’) are as follows: PHLPP1 (NM_194449.4): F: TGTGCCTGAGTGGGTATGTG, R: CATCAGAAGGTTAGGTGGGAG; PHLPP2 (NM_015020.3): F: TACCTGCCTTATCGTTTC, R: GAGTATTGCCGTCGCTTC; ACTB (NM_001101.5): F: GTCACCAACTGGGACGACAT, R: TAGCAACGTACATGGCTGGG. mRNA expression levels were quantified with ACTB as the reference gene. The PCR cycling conditions were as follows: 95 °C for 10 min; followed by 40 cycles at 95 °C for 15 s and 60 °C for 45 s; and a final extension step of 95 °C for 15 s, 60 °C for 1 min, 95 °C for 15 s and 60 °C for 15 s.

### Immunoblotting

Cells were lysed on ice with lysis buffer, and quantified using a Bicinchoninic Acid Protein Assay Kit (Beyotime Biotechnology). Equivalent quantities (25 µg) of protein lysates were separated by sodium dodecyl sulfate-polyacrylamide gel and then transferred electrophoretically to polyvinylidene fluoride membranes (Millipore). Once the transfer of the proteins was completed, the membranes were blocked with 5% skimmed-milk solution and incubated overnight at 4 °C with the primary antibody, PHLPP1 (PA5-34434, Invitrogen), PHLPP2 (ab227673, Abcam), β-actin (4970s, CST), AKT (4691s, CST), p-AKT (4060s, CST). Secondary antibodies were labeled with horseradish Peroxidase. Visualization was detected using an ECL detection system (Bio-Rad, CA, USA).

### Knockdown or overexpression of PHLPP1

Short hairpin interfering RNAs (shRNA) targeting human PHLPP1 (shPHLPP1-1: 5’-GGAAGACGCTGCTTCTGAA-3’; shPHLPP1-2, 5’-GAATGTATAATGTCCGTAA-3’; and shPHLPP1-3, 5’-GGCTGCGACAAGTCTCCAA-3’), and negative control (shNC, 5’-TTCTCCGAACGTGTCACGTTT-3’) were synthesized and constructed into lentiviral plasmids (pLKO.1). For overexpression, pLVX-puro lentiviral plasmid containing PHLPP1 (NM_194449.4) cDNA was used along with an empty vector as a control (oeNC). Transfection was performed using the Lipofectamine 2000 system. Subsequently, 48 h after transfection, recombinant vector in the cell supernatant was collected and transduced into KGN cells.

### Cell proliferation

Cell Counting Kit-8 (CCK-8; Dojindo, Japan) was used to detect cell proliferation. Briefly, KGN cells were planted into 96-well culture plate with a primary density of 3 × 10^3^ cells per well. At the specified time points, CCK-8 solution was added into the cells at 37 °C for 2 h incubation, and the absorbance was scanned by a microplate reader.

### Flow cytometry assay

The staining procedures followed two steps: (1) 20 min of incubation with Annexin-V-FITC at 4 °C and (2) another 20 min of incubation with propidium iodide (PI). A flow cytometer (BD Biosciences) was used to measure apoptosis.

### ROS production

Intracellular ROS accumulation was measured via 2’,7’-diclorodihydrofluorescein diacetate (DCFDA) staining. Briefly, KGN cells were incubated with 10 µM DCFDA in PBS for 25 min at 37 °C, and nuclei were stained with DAPI for 10 min. The cells were collected and analysed using a flow cytometer.

### Glycolysis assay

Extracellular acidification rate (ECAR) was measured with a XF24 Extracellular Flux Analyzer [27]. Briefly, cells digested to a density of 1 × 10^4^/well, were seeded in XF-24 culture plates (Agilent Technologies, Santa Clara, CA, USA), and were then placed in an incubator of 37 °C and 5% CO_2_ for 24 h. Around 1 h before detection, culture medium was replaced by XF Base Medium (Agilent Technologies). Subsequently, the cells were treated sequentially with 1 µM of glucose, 1 µM of oligomycin (ATP synthase inhibitor) and 0.5 µM of 2-DG (the glycolytic inhibitor) at time points for measurement of ECAR.

### Sample collections

This study was approved by the Ethics Committee of Affiliated Hospital of Inner Mongolia Minzu University. Informed consents were obtained from women ages 25 to 38 years old. Granulosa cells were collected from 30 PCOS women [15 hyperandrogenic PCOS (HA-PCOS), 15 normo-androgenic PCOS (NA-PCOS)] and 15 controls who had normal endocrine as previously described [28]. Demographic and clinical parameters of participants were listed in Table 1.

**Table 1:** Basic clinical features of participants

	Control	NA-PCOS	HA-PCOS
**Age**	31.9 ± 4.37	29.1 ± 3.20	31.1 ± 4.28
**BMI (kg/m** ^**2**^**)**	25.6 ± 2.63	26.5 ± 2.87	27.9 ± 4.06
**Ferriman-Gallwey Score**	3.13 ± 1.18	6.07 ± 1.33^a^	10.1 ± 1.49^a, b^
**Total testosterone (nmol/L)**	1.49 ± 0.21	2.03 ± 0.42^a^	3.16 ± 0.39^a, b^
**SHBG (nmol/lL)**	60.5 ± 2.20	54.1 ± 3.48^a^	45.4 ± 3.21^a, b^
**FAI**	2.46 ± 0.40	3.76 ± 0.83^a^	6.99 ± 0.99^a, b^
**DHEAS (µmol/L)**	3.46 ± 0.26	4.99 ± 0.25^a^	6.65 ± 0.32^a, b^
**17-OHP (ng/ml)**	0.93 ± 0.19	0.94 ± 0.32	1.10 ± 0.19
**FSH (mIU/ml)**	6.11 ± 0.26	5.91 ± 0.29	6.01 ± 0.32
**LH (mIU/ml)**	6.44 ± 0.31	8.47 ± 0.29^a^	11.4 ± 0.63^a, b^
**TSH (µIU/ml)**	2.05 ± 0.37	1.79 ± 0.28	1.91 ± 0.33

*BMI* Body mass index, *SHBG* Sex hormone-binding globulin, *FAI *Free androgen index, DHEAS Dehydroepiandrosterone sulfate, *17-OHP *17-Hydroxyprogesterone, *FSH *Follicularstimulating hormone, *LH* Luteinizing hormone, *TSH *Thyroid-stimulating hormone

^a^Significant difference between control and NA-PCOS or HA-PCOS subjects (*p* < 0.001)

^b^Significant difference between NA-PCOS and HA-PCOS subjects (*p* < 0.001)

### Statistical analysis

GraphPad Prism8.4.2 was used for data analysis. Data was expressed as mean ± SD. Comparisons between 2 groups were performed by T-test, comparisons among multiple groups were performed by one-way ANOVA. *p*-values < 0.05 indicate statistical significance.

## Results

### DHT treatment inhibited the proliferation and glycolysis activity in human KGN cells

**Fig. 1:**
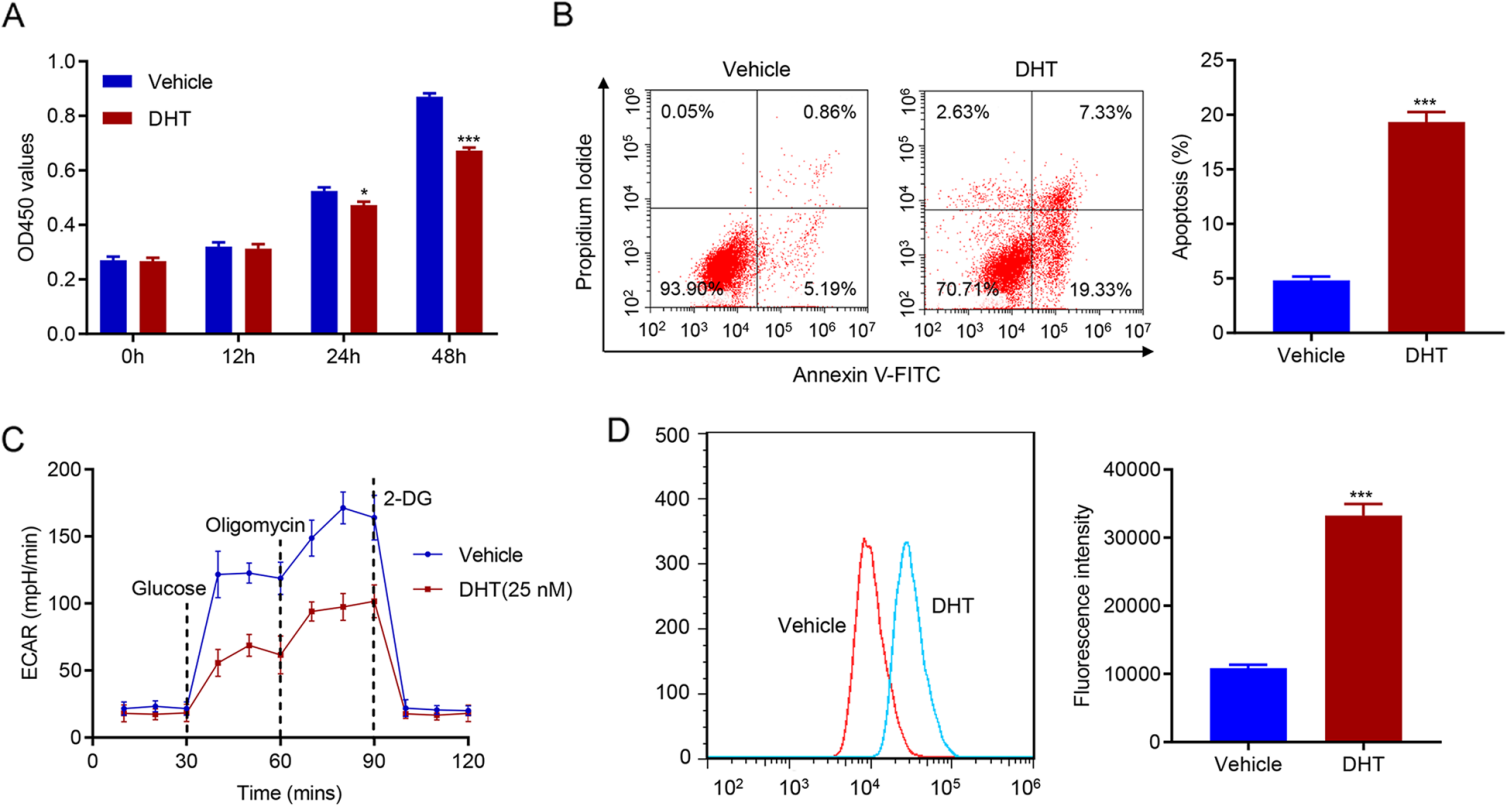
DHT treatment inhibited the proliferation and glycolysis activity in KGN cells. **A** Proliferation of KGN cells was deeply decreased after DHT treatment. **B** DHT treatment significantly increased apoptosis of KGN cells. **C** The glycolysis activity of KGN cells was suppressed by DHT treatment. **D** DHT treatment significantly promoted the production of ROS in human KGN cells. * *p* < 0.05, *** *p* < 0.001 vs. Vehicle

DHT was used to detect the action of high levels of androgen on proliferation and glycolysis activity in KGN cells. DHT treatment significantly suppressed KGN cell proliferation (Fig. 1A), and promoted the apoptosis of human KGN cells (Fig. 1B). DHT treatment also significantly inhibited glycolysis (Fig. 1C), and elevated ROS generation (Fig. 1D) in KGN cells. These data suggest that DHT treatment inhibited the proliferation and glycolysis of KGN cells.

### Knockdown of PHLPP1 ameliorated the DHT-induced injury in human KGN cells

**Fig. 2:**
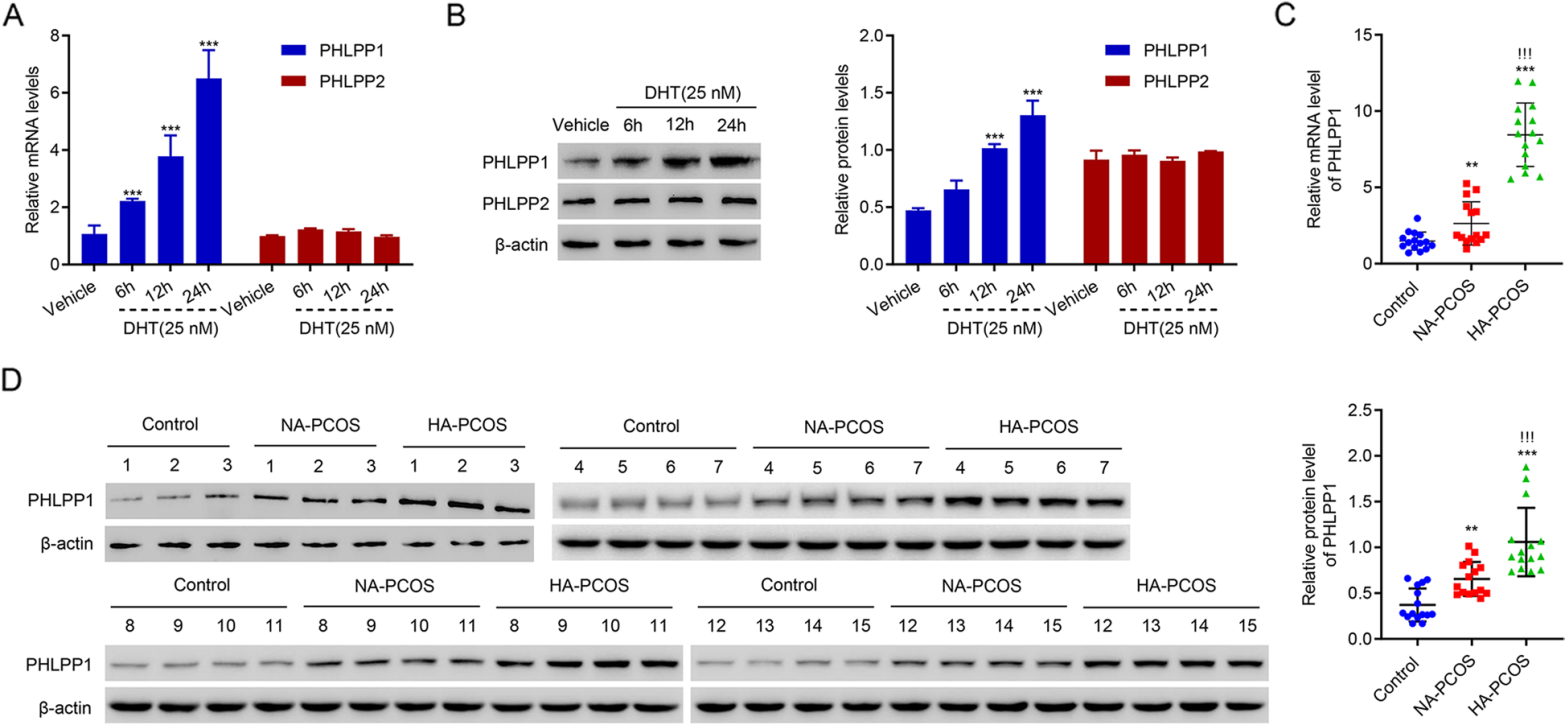
PHLPP1 is upregulated in DHT-treated KGN cells and patients with PCOS. **A**, **B** mRNA and protein levels of PHLPP1 and PHLPP2 in KGN cells after 6, 12 and 24 h of DHT treatment. **C**, **D** mRNA and protein levels of PHLPP1 in patients of the control, HA-PCOS, and NA-PCOS groups. ** *p* < 0.01,*** *p* < 0.001 vs. Vehicle or control. !!! *p* < 0.001 vs. NA-PCOS

**Fig. 3:**
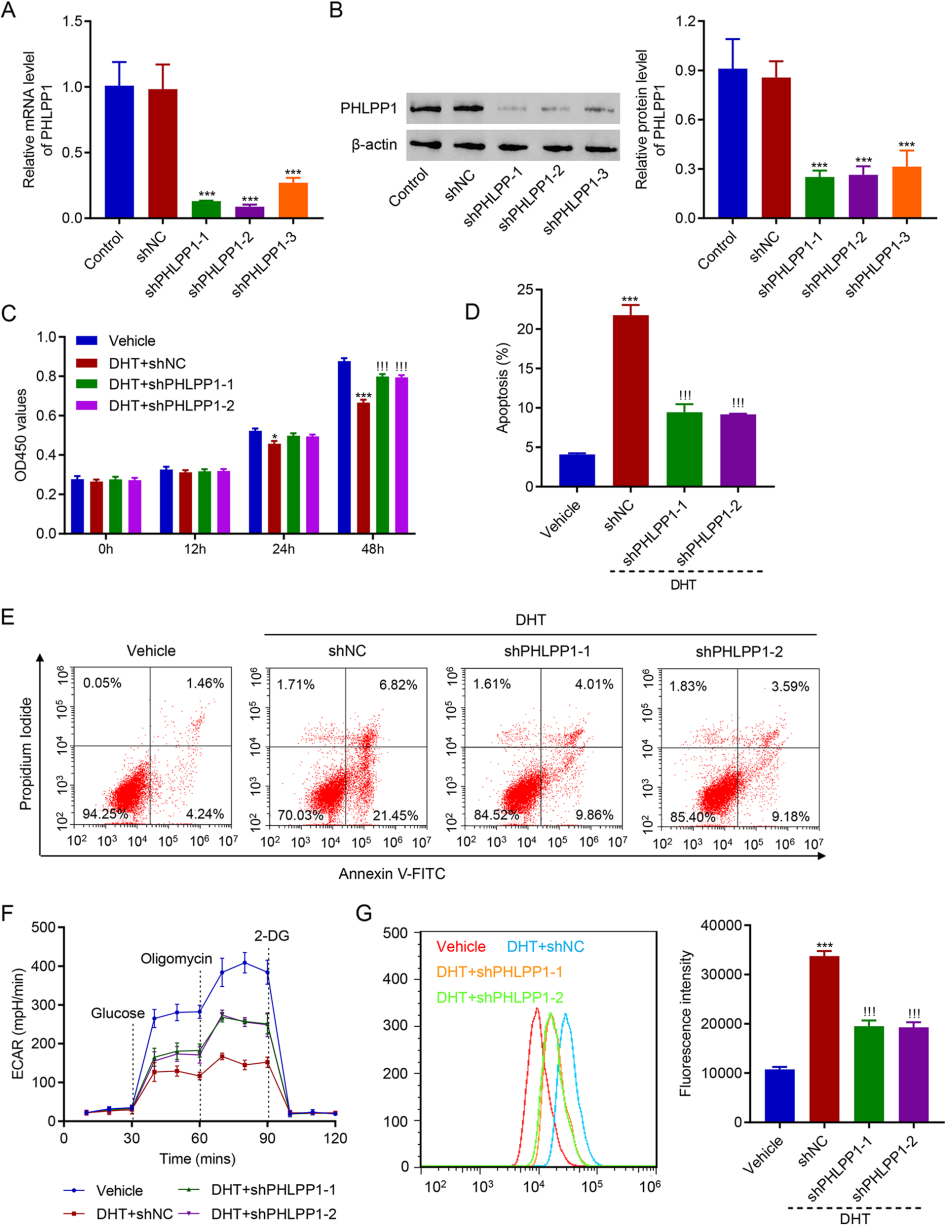
Knockdown of PHLPP1 ameliorated the DHT-induced damage of KGN cells. **A**, **B** PHLPP1 was successfully silenced. **C** Proliferation of DHT-treated cells was significantly promoted by knockdown of PHLPP1. **D**, **E** Silencing PHLPP1 inhibited apoptosis of DHT-treated KGN cells. **F** Silencing PHLPP1 promoted glycolysis in DHT-treated KGN cells. **G** ROS production in DHT-treated cells was suppressed by knockdown of PHLPP1. * *p* < 0.05, *** *p* < 0.001 vs. Vehicle or shNC. !!! *p* < 0.001 vs. DHT + shNC

As mentioned above, PHLPPs are important regulars of glycolysis and AKT. To figure out whether PHLPPs play a role in DHT regulation of cell proliferation and glycolysis, the expression of PHLPPs was examined after DHT treatment. Results showed that DHT treatment time-dependently up-regulated PHLPP1 at both mRNA and protein levels, but did not affect PHLPP2 (Fig. 2A and B). Similarly, it was found that the mRNA and protein levels of PHLPP1 were also increased significantly in the HA-PCOS subjects compared with the controls or NA-PCOS subjects (Fig. 2C and D). Therefore, PHLPP1 was focused in the subsequent study. To study the role of PHLPP1 in DHT regulation of cell proliferation and glycolysis, lentivirus was used to silence PHLPP1. Results showed that PHLPP1 was successfully silenced (Fig. 3A and B). More importantly, silencing PHLPP1 significantly ameliorated DHT-suppressed proliferation of KGN cells (Fig. 3C). DHT treatment promoted apoptosis of KGN cells, which was abolished by silencing of PHLPP1 (Fig. 3D and E). The inhibitory effect of DHT on glycolysis was significantly suppressed by PHLPP1 knockdown (Fig. 3F). ROS levels were significantly elevated by DHT treatment, but silencing of PHLPP1 remarkably decreased ROS levels in KGN cells (Fig. 3G). These results demonstrate that knockdown of PHLPP1 ameliorated the DHT-induced injury in human KGN cells.

### Inhibiting AKT rescued the effect of PHLPP1 silencing on cell proliferation and glycolysis in DHT-treated KGN cells

**Fig. 4:**
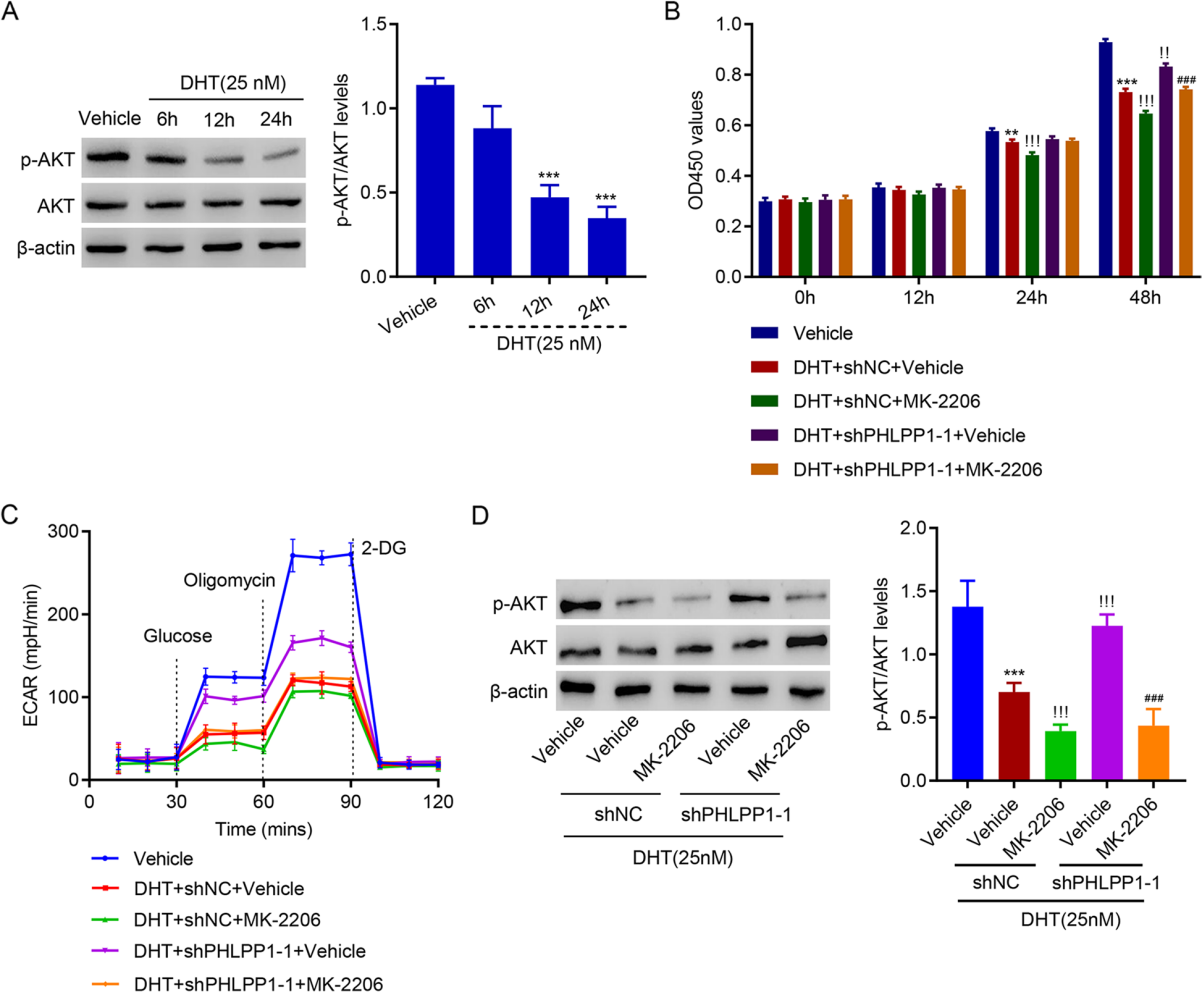
Inhibiting AKT rescued the function of PHLPP1 silencing in DHT-treated KGN cells. **A**, **B** Protein levels of AKT and p-AKT. B. AKT inhibitor MK-2206 abolished DHT-induced proliferation suppression in shPHLPP1-silencing KGN cells. **C** MK-2206 inhibited the glycolysis activity in DHT-treated PHLPP1-silencing KGN cells. **D** Levels of AKT and p-AKT. ** *p* < 0.01, *** *p* < 0.001 vs. Vehicle; !! *p* < 0.01, !!! *p* < 0.001 vs. DHT + shNC + Vehicle; ### *p* < 0.001 vs. DHT + shPHLPP1-1 + Vehicle

To figure out whether AKT signaling pathway play a role in DHT regulation of cell proliferation and glycolysis, the activation of AKT was examined after DHT treatment. Results showed that DHT treatment time-dependently down-regulated activation of AKT signaling pathway (Fig. 4A). To further investigate the role of AKT signaling, AKT inhibitor, MK-2206 was introduced. Administration of MK-2206 abolished DHT-suppressed proliferation of KGN cells with PHLPP1 silencing (Fig. 4B). MK-2206 also inhibited the glycolysis activity in DHT-treated PHLPP1-silencing KGN cells (Fig. 4C). Western blotting results indicated that MK-2206 significantly decreased levels of p-AKT induced by DHT and PHLPP1 silencing (Fig. 4D). Together, these results suggest that inhibiting AKT rescued the effect of PHLPP1 silencing on cell proliferation and glycolysis in DHT-treated KGN cells.

### AKT activator SC79 disrupted the effect of PHLPP1 overexpression on cell proliferation and glycolysis in KGN cells

**Fig. 5:**
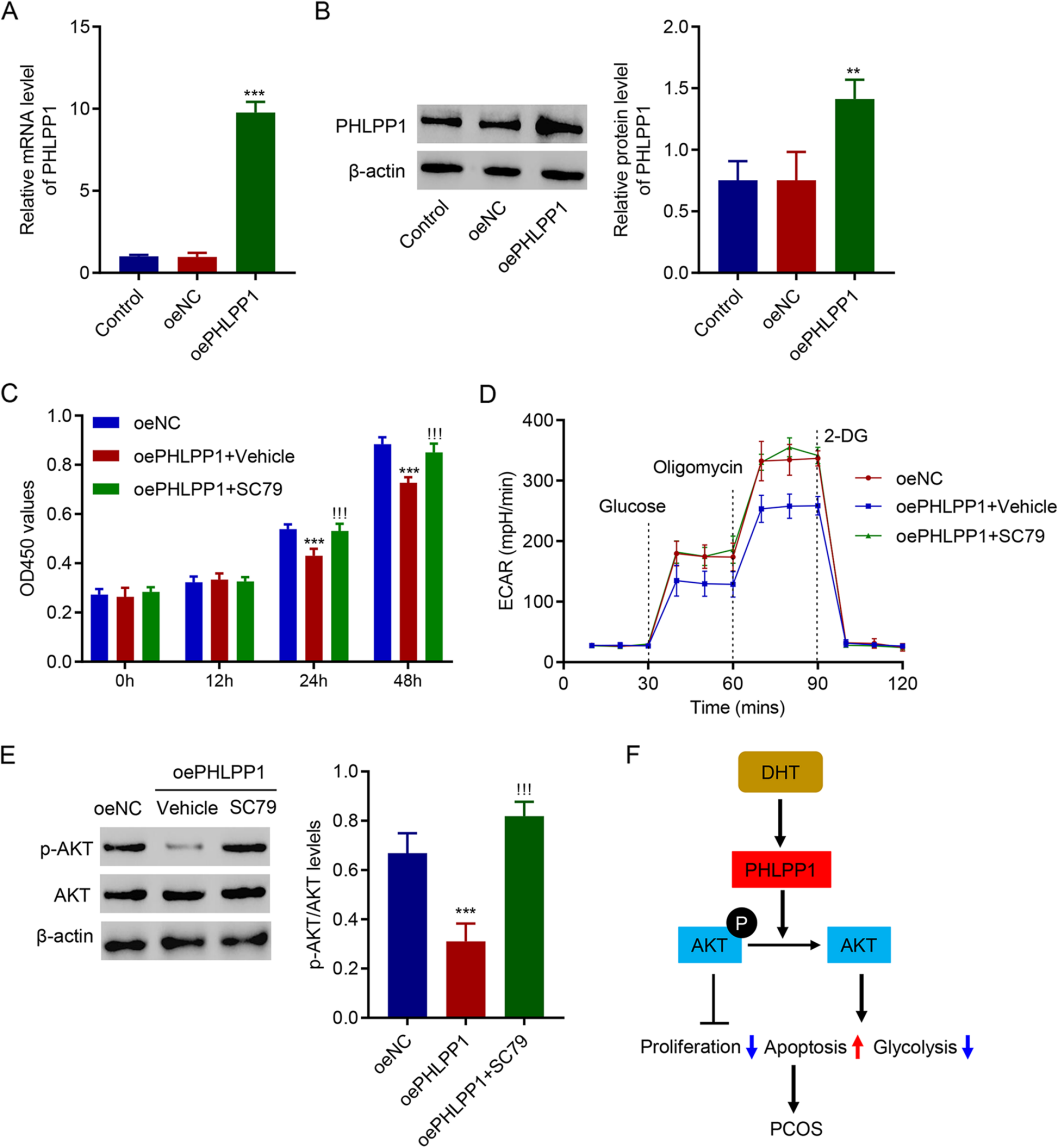
AKT activator SC79 disrupted the function of PHLPP1 overexpression. **A**, **B** PHLPP1 was successfully overexpressed. **C** SC79 promoted the proliferation of PHLPP1-overexpressing KGN cells. **D** SC79 promoted glycolysis of PHLPP1-overexpressing KGN cells. **E** Expression of AKT and p-AKT. **F** Schematic diagram of the mechanism by which PHLPP1 inhibits the growth and glycolysis of human ovarian granular cells through inactivating AKT pathway. ** *p* < 0.01, *** *p* < 0.001 vs. oeNC; !!! *p* < 0.001 vs. oePHLPP1 + Vehicle

To further study PHLPP1 and AKT, PHLPP1 was successfully overexpressed (Fig. 5A and B), and an AKT activator, SC79, was introduced. Results demonstrated that administration of AKT activator, SC79, significantly increased proliferation of KGN cells with PHLPP1 overexpression (Fig. 5C). Administration of SC79 also remarkably increased glycolysis activity of KGN cells with PHLPP1 overexpression (Fig. 5D). Western blotting results indicated that SC79 significantly increased level of p-AKT in KGN cells with PHLPP1 overexpression (Fig. 5E). These data suggest that the AKT activator, SC79, disrupted the effect of PHLPP1 overexpression on cell proliferation and glycolysis in KGN cells.

## Discussion

We demonstrated that DHT treatment suppressed proliferation, promoted apoptosis and ROS, and inhibited glycolysis of KGN cells, which were ameliorated by silencing PHLPP1. Mechanism study indicated that DHT-caused injury in PHLPP1-silencing cells was abolished by administration of AKT inhibitor. In contrast, administration of AKT activator disrupted the function of PHLPP1. For the first time, our study indicated that PHLPP1 mediates cell proliferation, apoptosis, ROS generation, and glycolysis of KGN cells by regulating AKT signaling pathway (Fig. 5F).

Converting glucose to pyruvate, glycolysis involves in a many bioprocesses [11]. Glycolysis plays a fundamental role in supporting cell growth and proliferation [12]. More importantly, it has been reported that perturbation of the expression of glycolytic enzymes was associated with infertility [16]. Zhao et al. have reported that enhanced glycolysis and inhibited tricarboxylic acid cycle were found in women with PCOS [17]. Here, we found that DHT administration resulted in significant damage of KGN cells through regulating PHLPP1 expression. Data demonstrates, for the first time, that silencing PHLPP1 ameliorated DHT-induced cell injury.

AKT plays a very important role in cell growth, division, and apoptosis [29]. AKT activation increases glycolysis [25]. Hojlund et al. have shown that AKT activation was significantly inhibited in PCOS skeletal muscle [30]. These data suggested that the status of AKT pathway might be tissue or cell-type specific. Our study indicated that inhibition of AKT rescued the role of PHLPP1 silencing in DHT-treated KGN cells, while activation of AKT disrupted the function of PHLPP1 overexpression. The findings suggest that PHLPP1 regulates glycolysis and cell proliferation through regulating AKT, and indicate that targeting AKT/PHLPP1 might provide a new direction for manipulating glycolysis and cell proliferation.

PHLPP1, first discovered by Gao et al., suppresses growth factor signaling through dephosphorylating Akt [20]. Overexpression of PHLPP1 has been shown to inhibit cell proliferation [23], while silencing of PHLPP1 has been shown to contribute to cell growth through regulating of AKT phosphorylation [23]. Studies also supported that AKT activation promoted glucose uptake and aerobic glycolysis [25, 26]. In the current study, we revealed a role of PHLPP1, showing that PHLPP1 suppressed glycolysis and promoted apoptosis of KGN cells through inhibition of AKT signaling pathway. Our study further showed PHLPP1 overexpression-caused cell injury could be abolished by administration of AKT activator, SC79. The results improved the understanding of the possible mechanism by which PHLPP1 regulates the proliferation and glycolysis of KGN cells. Previous studies have demonstrated that glycolytic enzymes are regulated by complex mechanisms, such as AKT, ERK, and NF-κB pathways [31–33]. The AKT pathway is responsible for glycolysis by regulating HIF-1α target genes ENO1 and LDHA [34, 35]. These data indicate that PHLPP1 may regulate ENO1 and LDHA expression via the AKT/HIF-1α signaling pathway. Xiong et al. demonstrated that PHLPP1 suppresses glycolysis via inhibiting Akt phosphorylation and mitochondrial localization of HK2, a key enzyme that determines the direction and magnitude of glucose flux [36]. However, whether other glycolytic enzymes and signlaing pathways are regulated by PHLPP1 should be further determined. It is worth to mention that only in vitro experiments and human ovarian granular KGN cells were included. Future in vivo studies and using primary cultured granulosa cells of women with PCOS may show more meaningful results. Although further studies are needed, this study identifies a new molecular mechanism by which PHLPP1/AKT/glycolysis regulates DHT-induced injury of human ovarian granular cells.

## Conclusions

Taken together, the results revealed a novel role of PHLPP1/AKT axis, indicating that knockdown of PHLPP1 ameliorated the DHT-induced injury in human KGN cells through regulation of AKT signaling pathway. The data highlighted the significance of PHLPP1/AKT axis which may benefit the treatment of PCOS.

## Data Availability

All data generated or analysed during this study are available from the corresponding author on reasonable request.

## Abbreviations

PCOS: Polycystic ovary syndrome
PHLPP1: PH domain Leucine-rich repeat Protein Phosphatase 1
DHT: Dihydrotestosterone
ECAR: Extracellular acidification rate
AKT: Protein kinase B
DCFDA: 2’,7’-dichlorofluorescein diacetate
